# Nanopore Sequencing and Hi-C Based De Novo Assembly of *Trachidermus fasciatus* Genome

**DOI:** 10.3390/genes12050692

**Published:** 2021-05-06

**Authors:** Gangcai Xie, Xu Zhang, Feng Lv, Mengmeng Sang, Hairong Hu, Jinqiu Wang, Dong Liu

**Affiliations:** 1 Institute of Reproductive Medicine, Medical School, Nantong University, Nantong 226001, China; sangmm12345@163.com; 2 Nantong Laboratory of Development and Diseases, School of Life Science, Nantong University, Nantong 226001, China; zhangxu0213@yeah.net; 3 Key Laboratory of Neuroregeneration of Jiangsu and Ministry of Education, Co-Innovation Center of Neuro-Regeneration, Nantong University, Nantong 226001, China; 4 Nantong College of Science and Technology, Qingnian Middle Road 136, Nantong 226006, China; prlf2019@163.com; 5 State Key Laboratory of Genetic Engineering, Institute of Genetics, School of Life Sciences, Fudan University, Shanghai 200438, China; hrhu@fudan.edu.cn

**Keywords:** *Trachidermus fasciatus*, genome assembly, novel gene, nanopore, Hi-C

## Abstract

*Trachidermus fasciatus* is a roughskin sculpin fish widespread across the coastal areas of East Asia. Due to environmental destruction and overfishing, the population of this species is under threat. In order to protect this endangered species, it is important to have the genome sequenced. Reference genomes are essential for studying population genetics, domestic farming, and genetic resource protection. However, currently, no reference genome is available for *Trachidermus fasciatus*, and this has greatly hindered the research on this species. In this study, we integrated nanopore long-read sequencing, Illumina short-read sequencing, and Hi-C methods to thoroughly assemble the *Trachidermus fasciatus* genome. Our results provided a chromosome-level high-quality genome assembly with a predicted genome size of 542.6 Mbp (2*n* = 40) and a scaffold N50 of 24.9 Mbp. The BUSCO value for genome assembly completeness was higher than 96%, and the single-base accuracy was 99.997%. Based on EVM-StringTie genome annotation, a total of 19,147 protein-coding genes were identified, including 35,093 mRNA transcripts. In addition, a novel gene-finding strategy named RNR was introduced, and in total, 51 (82) novel genes (transcripts) were identified. Lastly, we present here the first reference genome for *Trachidermus fasciatus*; this sequence is expected to greatly facilitate future research on this species.

## 1. Introduction

The roughskin sculpin (*Trachidermus fasciatus*) is a small, carnivorous, and catadromous fish that has been found across the coastal areas of China, Korea, and Japan [1,2]. Historically, roughskin sculpin has been named one of the four most famous fishes in China and has been treated as a valuable food source by the Chinese population [3]. However, the population size of this fish species has declined significantly during the past decades due to overfishing and environmental changes in spawning and habitat site [1,4]. Since 1988, roughskin sculpin has been listed as a Class II protected animal by the Chinese government, which encouraged the development of a farming system for its domestication [5]. It had been reported that the karyotype of this species is 2*n* = 40 ([6]), and more recently, the genetic diversity [7] and genomic signature [8] of *Trachidermus fasciatus* had also been studied, which might be important for its conservation management. A reference genome of this species is important for both the development of a farming system and future genetic studies. Although its mitochondrial genome has been previously identified [9], the nuclear genome sequence is still unavailable. 

During the past decades, high-throughput DNA sequencing technologies have advanced significantly, including Illumina short-read sequencing, Pacific biosciences, and Oxford nanopore long-read sequencing [10,11]. It has been shown that the nanopore long-read sequencing technology can be used for genome assembly in different species, including bacterial [12], human [13,14], and rice [15]. Furthermore, chromosome-scale assemblies of human and mouse genomes can be generated by integrating short-read DNA sequencing and Hi-C chromatin interaction mate-pair sequencing [16]. By combining Hi-C and short-read data, a scaffold spatial orienting accuracy of 99% was achieved [16].

**Figure 1:**
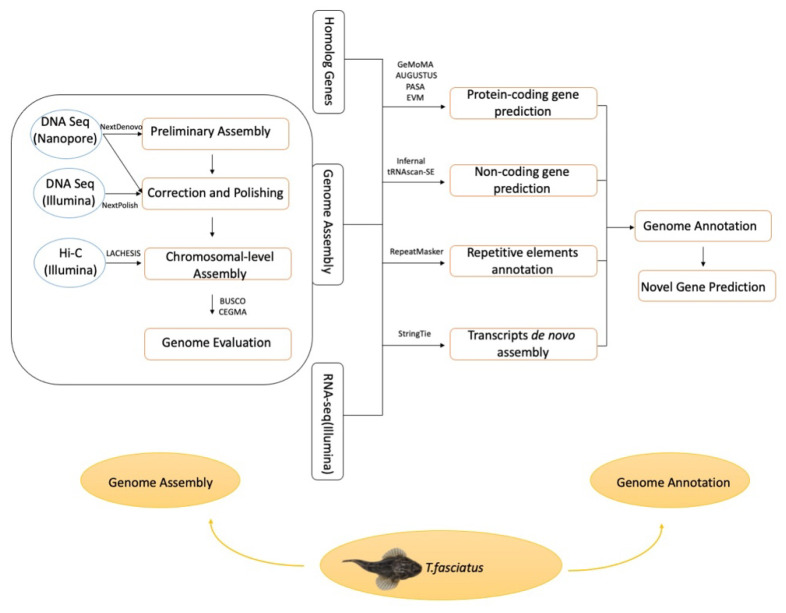
Pipeline of *Trachidermus fasciatus* genome assembly and annotation.

In this study, Oxford nanopore sequencing, Illumina short-reads sequencing technologies, and the Hi-C method were integrated for de novo genome assembly of *Trachidermus fasciatus* (Figure 1). This study not only provides the first high-quality reference genome for the communities studying roughskin sculpin but also identified 51 novel genes and 82 novel transcripts.

## 2. Materials and Methods

### 2.1. Tissue Extraction and Sequencing

A single 1 year old live *Trachidermus fasciatus* (NCBI:txid290630) fish was collected for tissue extraction. The study was conducted following Chinese law for the Protection of Animals, and the animal was treated properly and in line with the ARRIVE guidelines. Tissues from seven different areas were collected for RNA sequencing, including liver, gall bladder, stomach, heart, kidney, gill, and skin. The muscle samples were collected for both of DNA sequencing (nanopore and Illumina sequencing) and Hi-C sequencing (Illumina sequencing). The tissue samples were stored in liquid nitrogen before sequencing. *Trachidermus fasciatus* genomic DNA was extracted using the QIAGEN® Genomic DNA extraction kit (Cat#13323, Qiagen, Valencia, CA, USA) following manufacturer’s instructions. An RNA sequencing library was constructed using the Illumina TruSeq RNA library preparation kit. Nanopore sequencing was carried on a Nanopore GridION X5/PromethION sequencer (ONT, Oxford, UK), while Illumina sequencing (DNA, RNA, Hi-C) was performed using the Illumina HiSeq platform (Illumina, San Diego, CA, USA). Finally, the Hi-C library preparation was performed according to a previously reported protocol [17].

### 2.2. De Novo Genome Assembly

NextDenovo was used for genome assembly, including sequencing error correction, preliminary assembly, and genome polishing. The NextCorrect module was used for raw read correction and consensus sequence extraction. The NextGraph module was used for preliminary assembly, and the NextPolish module was used for genome polishing [18]. At the genome polishing stage, nanopore reads were used repetitively three times, and Illumina sequencing reads were used four times for genome correction. The seed cutoff was set at 38 Kbp, and the read cutoff was set at 1 Kbp for the NextDenovo genome assembly, while default parameters were used for other settings.

### 2.3. Quality Evaluation of Genome Assembly 

To evaluate the quality of the genome assembly, four evaluation metrics were applied, including genome completeness, genome accuracy and consensus, GC proportion and sequencing-depth distribution (GC-depth analysis), and genome contamination. Both BUSCO [19] and CEGMA [20] were used for genome completeness evaluation. BUSCO evaluated the completeness of the assembly by matching it with the ortholog genes from the OrthoDB [21] database (actinopterygii_odb9), and the evaluation of CEGMA was performed by comparing the evolutionarily conserved core protein-coding genes in eukaryotes (248 core genes). To assess the assembled genome sequence accuracy and consensus, Illumina sequencing reads were mapped onto the genome by Burrows–Wheeler aligner (BWA) [22]. Samtools and bcftools [23] were used for SNP and Indel calculations. The percentage of homogeneous SNPs was considered as the single nucleotide error rate of the assembled genome. For the GC-depth analysis, nanopore sequencing reads were mapped onto the genome assembly using minimap2 [24], and the GC content proportion and long read coverage were calculated for each sliding window (size of 10 kbp) of the assembled genome. Finally, the assembled genome was compared to the sequences from nucleotide sequence database (NT, ftp.ncbi.nih.gov/blast/db) to examine the interspecies contamination. The genome was divided into 1 Mbp bins and then aligned with the NT sequences using the blastn [25] software. The mapping statistics were summarized based on the results from each bin.

### 2.4. Hi-C Guided Genome Assembly

The raw paired-end Hi-C reads were preprocessed by fastp [26] for adapter trimming and low-quality read filtering (only keeping reads with Phred Score > 15, and 5 > number of Ns in the reads). Each pair of clean reads was mapped onto the assembled genome using bowtie2 [22] (version: 2.3.2, parameters: -end-to-end, --version-sensitive -L 30). For the reads that could be mapped onto the genome, the DpnII restriction endonuclease recognition sequence pattern GATC was searched, and the reads were cut at their restriction sites and used for further mapping. Each pair of uniquely mapped reads was merged for further analysis. LACHESIS [16] (parameters: CLUSTER MIN RE SITES = 100; CLUSTER MAX LINK DENSITY = 2.5; CLUSTER NONINFORMATIVE RATIO = 1.4; ORDER MIN N RES IN TRUNK = 60; ORDER MIN N RES IN SHREDS = 60) was used to obtain chromosome-level scaffolds based on the primary assembly and the Hi-C read mapping information. To draw inter- and intra-chromosomal Hi-C interaction heatmap, each chromosome was divided into bins with length of 100Kbp, and coverage of Hi-C reads pairs for each pair of bins was calculated and treated as the linkage intensity. The linkage intensity for each bin pair was further natural log transformed for heatmap representation. 

### 2.5. Gene and Repetitive Element Annotation

Based on the RNA sequencing data from eight tissues, the assembled genome, and public homolog protein sequences, *Trachidermus fasciatus* genome was annotated at different levels, including its repetitive elements, non-protein-coding RNAs (ncRNA), and protein-coding genes. First, RepeatMasker [27] was applied to annotate the repetitive elements (RE), and the repeats masked genome was further used for gene annotation. To construct RE database for RepeatMasker, MITE-hunter [28] was used to search miniature inverted-repeat transposable elements (MITE DB) in reference assembly. The reference assembly was hard masked by MITE DB through RepeatMasker, and then RepeatModeler was used to search RE sequences in de novo (RM DB) based on MITE hard masked reference assembly. In addition, the MITE DB, RM DB, and Repbase [29] (Repbase-derived RepeatMasker libraries RELEASE 20181026) were combined into one repetitive elements database (RE DB), which was further used for RepeatMasker prediction of REs. For the protein-coding gene annotation, at the first stage, EVM [30] was used to integrate the annotation results from three methods, including transcriptome prediction by PASA [31], homolog protein predicted by GeMoMa [32], and de novo gene predicted by AUGUSTUS [33]. At the second stage, transcripts were assembled by StringTie by sequentially five procedures. Firstly, RNA-seq reads were mapped onto *Trachidermus fasciatus* genome by hisat2 [34] (Table 1 for mapping statistics), and then the primary transcripts were assembled by StringTie [35] without guide reference annotation for each tissue individually. Thirdly, the GTF files from the transcripts assembly of each tissue were merged by StringTie. Fourthly, the transcripts were assembled by StringTie with merged GTF file as guide reference annotation for each tissue individually. Finally, the GTFs from the second round of transcripts assembly were merged as StringTie transcripts. At the third stage, the transcripts from EVM integration and StringTie assembly were further merged by StringTie, and the coding regions of EVM-StringTie merged transcripts were predicted by TransDecoder, where the length of protein amino acids was required to be longer than 100. The coding sequences were further filtered by Gffread [36], where the transcripts lacking initial start codon, the terminal stop codon, or having an in-frame stop codon were discarded. Furthermore, Infernal [37] and tRNAscan-SE [38] were used to predict ncRNAs and tRNAs, respectively.

### 2.6. Novel Transcripts/Gene Discovery

RNR method (RefSeq-NT-RNASeq integrated novel gene selection) was created to identify novel genes and transcripts in *Trachidermus fasciatus*, which contains following three steps: model organism RefSeq sequence alignment, NCBI nucleotide database (NT) alignment, and RNA-Seq expression filtering. At model organism sequence alignment step, the RefSeq RNA sequences of four model organisms (*Homo sapiens*, *Mus musculus*, *Danio rerio*, and *Caenorhabditis elegans*) were downloaded from UCSC Genome Browser (hg38, mm10, danRer11, and ce11). The EVM-StringTie mRNA sequences were aligned to RefSeq RNA sequences by pblat [39] (same as blat but with multithreads support) with the setting of “-threads=40 -minScore=30 -minIdentity=60”. At the second step, the nucleotide database (NT) was downloaded from NCBI (ftp.ncbi.nlm.nih.gov:/blast/db/FASTA/nt.gz), and then the index of NT was built by “makeblastdb” (from NCBI-BLAST [40]). The mRNA sequences failed to be mapped at the first step were re-aligned onto NT database by blastn (NCBI-BLAST, value was set to be 0.05). At the last step, transcript-level expression values were calculated by salmon [41] software, and then only the unmapped transcripts from the first two steps with at least 50 reads detected in at least one tissue were defined as the final novel transcripts. Novel genes were defined as the genes that are associated with the novel transcripts at the final step and also with all of their transcripts unmapped at the first two steps. To study the protein sequence novelty, the predicted protein sequences of novel transcripts were aligned to NCBI NR database by blastp [42] (expected threshold 0.05). For the visualization of the novel genes, ggsashimi [43] was used to generate sashimi plot to show both of RNA-seq reads density and exon–exon junctional linkages on the candidate novel genes.

### 2.7. Data Availability and Software Details

The genome sequence and genome annotation of *Trachidermus fasciatus* has been stored in the Genome Warehouse of National Genomics Data Center [44], Beijing Institute of Genomics (BIG), Chinese Academy of Sciences, under accession number GWHACFF00000000.2, which is publicly accessible at https://bigd.big.ac.cn/gwh. R was used for graphical plotting, and the source and version of the software used are listed in Table 2 below. 

## 3. Results

### 3.1. Sequencing Data Sets

Following nanopore sequencing (Table 3), we obtained approximately 4 million reads that passed quality control, containing more than 87 billion nucleotide bases. The longest read was over 240 kbp, and the N50 was approximately 30 kbp. More than 70% of the reads had a length exceeding 10 kbp, while roughly 12% of the reads were exceeding 40 kbp. For Illumina sequencing, we obtained more than 350 million reads for both genome and Hi-C sequencing (Table 3), which contains more than 50 billion nucleotide bases. For RNA-seq of seven tissues, 434 million paired-end reads were sequenced, containing more than 65 billion bases. Based on the estimated assembly size, nanopore sequencing and Illumina sequencing (genomic DNA) had average genomic coverage of 161X and 100X, respectively (Table 3).

### 3.2. De Novo Assembly and Genome Polishing

*Trachidermus fasciatus* genome was preliminarily assembled based on nanopore sequencing data and then polished based on both of nanopore and Illumina sequencing data (Figure 1). In the preliminary stage, 62 contigs were assembled, with an N50 exceeding 23 Mbp. The longest contig was longer than 35 Mbp, while the total length of the preliminary genome was approximately 539.1 Mbp (Table 4). After polishing with the Illumina short-read sequencing data, the N50 increased from 23.4 to 23.55 Mbp, and the full-length assembly reached roughly 542.6 Mbp. Based on N90 information (Table 4), 23 contigs have a combined assembly coverage of 90%, which indicates that the number of N90 contigs (23) is close to the haploid chromosome number of *Trachidermus fasciatus* based on karyotype (2*n* = 40) reported previously [3,6].

### 3.3. Genome Quality Evaluation

**Figure 2:**
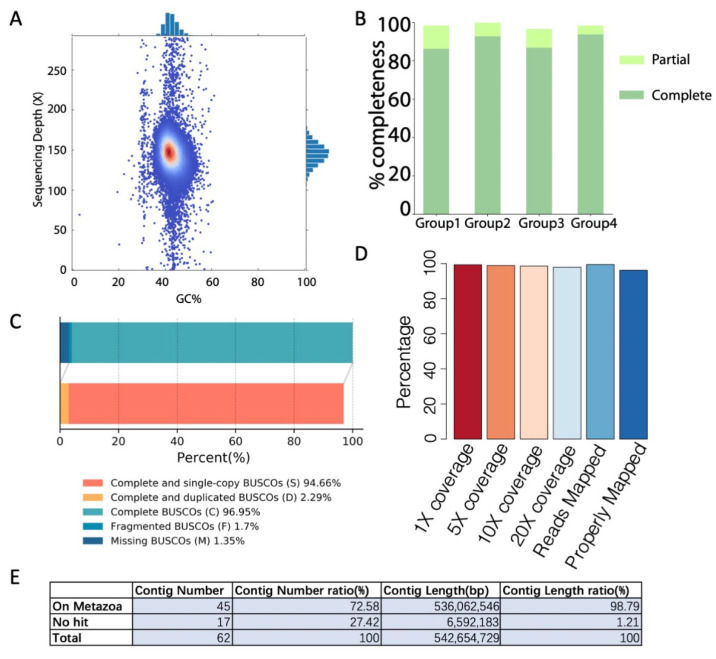
Quality evaluation of genome assembly. (**A**) GC content and sequencing depth distribution. (**B**) CEGMA evaluation. (**C**) BUSCO evaluation. (**D**) Illumina read mappability and genome coverage. The assembled genome is 99.35% covered at least once by Illumina reads, and 97.93% is covered at least 20 times. In addition, 99.48% of the Illumina reads were mapped to the assembled genome, and 96.22% were properly mapped (mapped paired reads with flag 0 × 2 set). (**E**) Genome contamination evaluation.

The quality of the assembled genome was further evaluated using different methods. First, the GC content and nanopore sequencing depth distribution were examined based on 10 kbp sliding windows. As shown in Figure 2A, only one peak was observed for the GC content and sequencing depth distribution, which indicates no interspecies contamination. Then, the completeness of the assembled genome was evaluated by both CEGMA [20] and BUSCO [19] (Figure 2B,C), and the results indicated a high percentage of completeness (98.39% and 96.95%, respectively). Next, high-quality Illumina sequencing reads were mapped onto the assembled genome to evaluate its quality, and the results revealed 99.48% reads mappability with 99.35% of the assembled genome being covered at least once (Figure 2D). Finally, genome contamination was examined by matching the assembled contigs with known metazoa genome sequences (based on 50 kbp bins). As shown in Figure 2E, 98.79% of the genome length can be matched with known metazoan genome sequences, which indicates no significant contamination from bacteria.

The single-base-level accuracy was examined by mapping the Illumina short reads onto the assembly. At a depth of no less than 5× genome-level coverage, 3444 homogeneous single nucleotide polymorphisms (SNPs) and 14,558 insertions or deletions (Indels) were found, occupying 0.000635% and 0.002683% of the genome, respectively. This gives the assembled genome with an estimated single-base accuracy of 99.9967%.

### 3.4. Hi-C Proximity-Guided Assembly of Chromosome-Level Scaffolds

**Figure 3:**
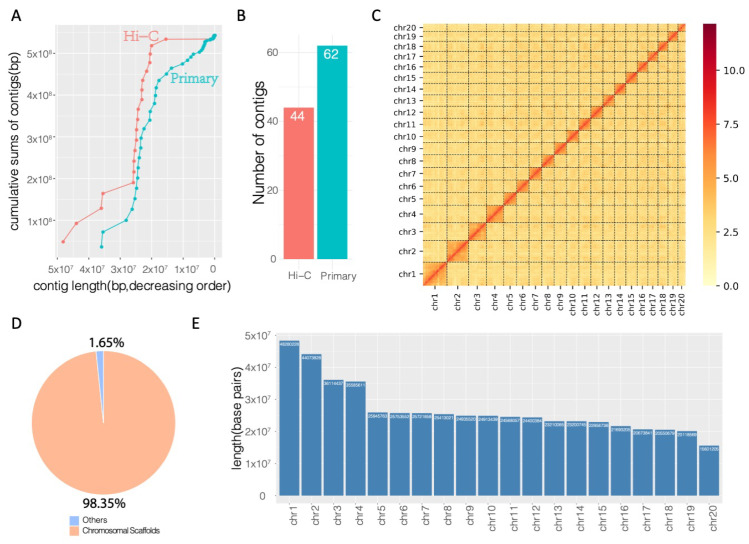
Hi-C enhanced genome assembly. (**A**) Comparison of cumulative sums of the contigs between the primary assembled genome and the Hi-C enhanced genome. (**B**) Reduced number of scaffolds/contigs in Hi-C enhanced genome. (**C**) Chromosomal level all-by-all Hi-C interaction heatmap (legend value is the natural log transformed linkage intensity). (**D**) Genomic percentage of the chromosome-level scaffolds. (**E**) Length distribution for each chromosome.

Compared to the primary assembly comprised of 62 contigs, the Hi-C-enhanced assembly showed longer cumulative scaffolds (Figure 3A). Using Hi-C chromatin interaction data, the contigs were rearranged based on the interaction information, and a total of 44 scaffolds were assembled (Figure 3B, Table S1). Based on the top 20 longest scaffolds, which represent the main chromosomal scaffolds of *Trachidermus fasciatus*, Hi-C chromatin interaction events were significantly enriched in intrachromosome but not interchromosome regions (Figure 3C). The number of N90 contigs/scaffolds decreased from 23 to 18 (Table S1), and the top 20 scaffolds reached a cumulative length of approximately 533.7 Mbp, occupying 98.35% of the assembled genome (Figure 3D). This indicates that a chromosome-scale genome assembly was achieved after Hi-C proximity-guided enhancement. The length in base pairs of each chromosome was illustrated by the barplot in Figure 3E, which shows that the longest chromosome (chr1) of *Trachidermus fasciatus* has about 48 million base pairs, and the length of shortest chromosome (chr20) is about 15.6 Mbp.

### 3.5. *Trachidermus fasciatus* Genome Annotation

**Figure 4:**
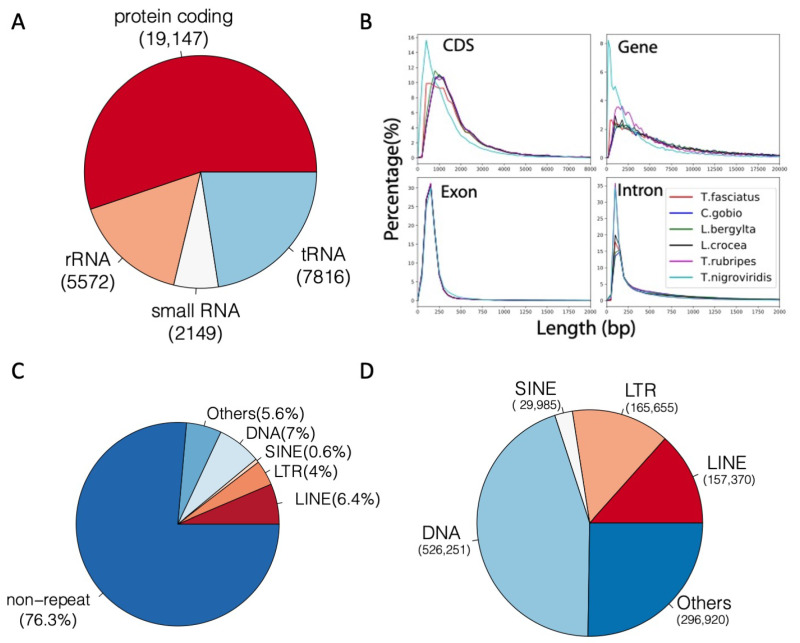
Summary of genome annotation for *Trachidermus fasciatus***.** (**A**) Number of protein-coding and non-protein-coding genes. (**B**) Gene annotation length distribution compared between close species. (**C**) Percentage of genome occupied by repetitive elements. (**D**) Number of repetitive elements in each class.

We integrated de novo and RNA sequencing-based annotation methods for *Trachidermus fasciatus* genome. Based on PASA [31] gene structure annotation, the AUGUSTUS [33] de novo protein-coding gene prediction, and GeMoMa [32] homolog gene prediction, 14,238; 25,741; and 22,211 genes were identified, respectively. Evidence Modeler (EVM) [30] was used to integrate the genes from these three methods, and 23,191 protein-coding transcripts were finally identified by EVM. For transcript assembly, 35,392 transcripts of 17,867 genes were assembled by StringTie [35]. By integrating the results from EVM and StringTie, 19,147 protein genes with 35,093 transcripts (mRNAs) were found, which were used as final annotation for the protein-coding genes of *Trachidermus fasciatus*. In addition to protein-coding genes, 5572 rRNA, 2149 small RNA, and 7816 tRNA non-protein-coding genes were identified (Figure 4A). Compared with the genomes of the five closest species of *Trachidermus fasciatus* with an available annotated genome, no abnormal length distribution was observed for CDS, genes, exons, and introns (Figure 4B). Furthermore, the repetitive elements were also annotated. In total, 23.7% of the *Trachidermus fasciatus* genome was covered by repetitive elements, including LTR (4%), LINE (6.4%), SINE (0.6%), and DNA repeats (7%) (Figure 4C). There are approximately 157,000 LINE elements, 526,000 DNA elements, 166,000 LTR elements, and 30,000 SINE elements (Figure 4D).

### 3.6. Novel Genes and Transcripts Identified

**Figure 5:**
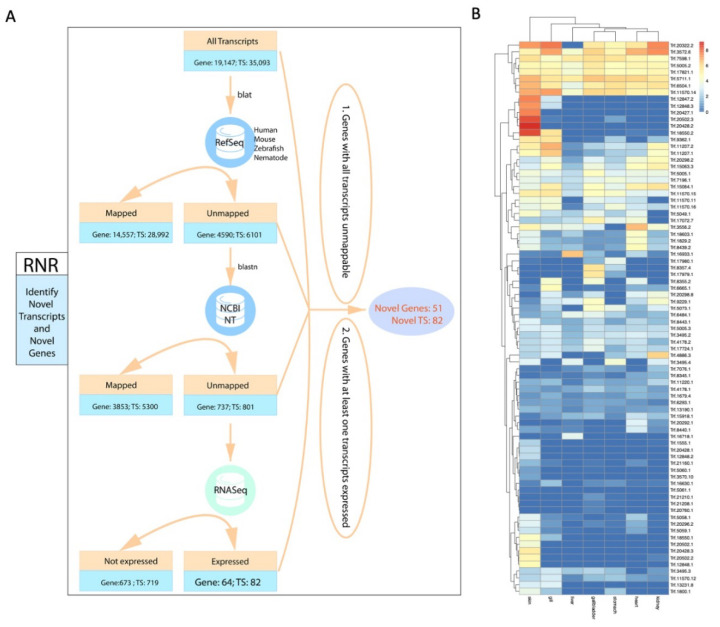
Identification of novel genes and transcripts. (**A**) Flowchart of RNR novel gene/transcript identification method. (**B**) Heatmap of the expression (log1p(FPKM)) of the novel transcripts in seven tissues.

As the first genome assembly for *Trachidermus fasciatus*, novel genes and transcripts were identified through RNR (RefSeq-NT-RNASeq integrated novel gene selection) strategy (Figure 5A). At the first step, transcript sequences were aligned onto the RefSeq sequences of four model organisms: human, mouse, zebrafish, and nematode. Among 35,093 protein-coding transcripts identified in this study, 6101 transcripts were failed to be aligned to RefSeq sequences of any selected organism. At the second step, a larger nucleotide sequence database (NCBI nucleotide database, or NT database), containing 63,454,572 sequences (genome and transcript sequences) from GenBank, EMBL, DDBJ and NCBI WGS, was used to find novel transcripts. Among 6101 transcript that failed to be mapped to any RefSeq sequence of selected organism, 5364 transcripts can be aligned to NT sequences, and 737 transcripts were unmappable to any of NT sequence. At the third step, detected RNA-seq reads (>50 reads) were further required to select novel transcripts, which led to 82 transcripts defined as novel transcripts. Those 82 transcripts were associated with 64 genes, and among those genes, 51 were defined as novel genes based on two criteria: firstly, all the transcripts of those genes should be unmappable to either RefSeq sequences or NT sequences, and secondly, at least one of their transcripts had detected RNA-seq reads (>50 reads). 

Next, the RNA-seq expression levels were examined for 82 novel transcripts identified in this study (Figure 5B). Based on the expression profile of the seven tissues (liver, gall bladder, stomach, heart, kidney, gill, and skin), some novel transcript showed tissue-specific expression pattern. Among those tissue-specific expressed novel transcripts, the skin-specifically-expressed novel gene Trf.20428 was studied in detail. Trf.20428 contains three isoforms, and all of them were found to be as novel transcripts. As illustrated in the Sashimi plot based on the RNA-seq bam files (Figure S1), Trf.20428 showed specific expression in skin compared to other tissues studied, and the intron-exon boundaries of this transcript can be clearly identified by the mapped reads. 

Furthermore, the protein sequences of the novel transcripts were mapped to NCBI NR database by blastp [42], and the protein sequences of 36 novel transcripts cannot be aligned to NR protein sequences under blastp expected threshold 0.05 (Figure S2A). For the 46 protein sequences that have mapped hits in the NR, the majority of them have an identity percentage with their best hits less than 60% (Figure S2B). The RNA-seq mapping detail for one of the blastp unmappable gene named Trf.5711 (with one transcript Trf.5711.1) was illustrated in Figure S3, which was highly expressed in all seven selected tissues. In summary, 82 transcripts and 51 genes (Tables S2 and S3, and sequence information in Supplementary File 1) from *Trachidermus fasciatus* were identified as novel transcripts and genes, respectively, and 36 novel transcripts contained protein sequences with no matched known sequences.

## 4. Discussion

In this study, we provided the first complete genome assembly for *Trachidermus fasciatus* (roughskin sculpin). Due to overfishing and environmental destruction, the population of roughskin sculpin is currently under threat in China, even though it has long been listed as a Class II protected animal. Our study might be important for future research on the protection and domesticated culturing of *Trachidermus fasciatus*. We not only provided the first genome reference for this species but also predicted its gene structures and annotated a total of 19,147 protein-coding genes. Genome annotation is important for future genetic studies on roughskin sculpin, which might provide a rich genetic resource for phenotypical and ecological studies. In addition to the genome assembly and genome annotation, in this study, we created a new method named RNR that can be used to identify novel transcripts/genes for a new species. Notably, we found 51 novel genes and 82 novel transcripts, which is useful to expand our knowledge about current gene pools from different species. 

At the stage of preliminary assembly, we got 62 contigs with a combined length of 539,115,043 bp. Multiple factors could contribute to the small number of contigs at this stage: firstly, the estimated genome size of the species *Trachidermus fasciatus* is relatively smaller compared to the species such as zebrafish (1679M, GRCz11/danRer11), mouse (2730M, GRCm38/mm10), and human (3257M, GRCh38/hg38); secondly, the sequencing depth for both of Illumina sequencing and Nanopore sequencing is quite high (100× and 160× respectively, Table 3); thirdly, the average length of nanopore reads is 21,384 bp, which could facilitate the assembly of contigs. Furthermore, based on the relationship (Table S4) between estimated chromosomes and the contigs of preliminary assembly (polished), the longest contigs from preliminary assembly occupies 99.798% of the estimated chromosome (chr3), which suggested that some of the preliminary contigs have achieved chromosome-level assembly.

To evaluate the accuracy of the assembled genome at single-nucleotide level, we mapped the Illumina sequencing reads onto the assembled genome and calculated the accuracy based on the proportions of SNPs and INDELS from the mapping results. However, this naïve method still has a few limitations: we did not consider the sequencing errors generated by Illumina sequencing method and also ignored the mapping errors (especially for repetitive elements).

RNR method proposed in our study contains three levels of gene filtering, model organism RefSeq filtering, NCBI NT database filtering, and RNA-seq expression level filtering. Although it is possible to directly map our target sequences onto NCBI NT database, it will be computationally time consuming due to a large size of NT database (63 million sequences). In RNR, we suggest mapping the target sequences onto selected model organisms (with better genome assembly and gene annotation compared to nonmodel organisms), which would greatly reduce the number of target sequences for NT mapping. 

In the previous study, the mitochondrial genome of *Trachidermus fasciatus* had been assembled [9]; thus, our study mainly focused on the nuclear genome assembly. To examine the mtDNA contamination in the nuclear genome assembly, we used blat (default setting) to map mtDNA sequence (NCBI Reference Sequence: NC_018770.1) onto our nuclear genome assembly, and no hit was found, which indicates low possibility of mtDNA contamination to our nuclear genome assembly.

At last, although we made the genome assembly and gene annotation publicly available, the genome browser tools to navigate the genome of *Trachidermus fasciatus* are still lacking. In the future, we will develop a genome browser to improve the accessibility of the genome-wide information for this species.

## 5. Conclusions 

In this study, through combining nanopore sequencing and Hi-C technologies, we assembled the first chromosomal-level high quality genome assembly for *Trachidermus fasciatus.* Furthermore, we proposed RNR method for novel genes finding, and in total 51 genes of *Trachidermus fasciatus* annotated in this study were identified as novel genes. Our study could greatly facilitate future studies for *Trachidermus fasciatus*.

## Figures and Tables

**Table 1:** Hisat2 mapping statistics for the RNA-seq datasets of seven tissues.

Sample	Number of Bases	Total (Pairs of Reads)	Mapped (Unique)	Mapped (Multiple)	Mapped (All)	Mapped (Percentage)
skin	8,250,236,700	27,500,789	22,963,898	2,672,115	25,636,013	93.22%
stomach	10,785,480,600	35,951,602	26,050,112	7,422,957	33,473,069	93.11%
gill	8,196,614,400	27,322,048	23,854,282	1,804,852	25,659,134	93.91%
gallbladder	10,626,212,100	35,420,707	30,789,079	1,382,971	32,172,050	90.83%
kidney	9,276,090,300	30,920,301	27,633,067	1,376,568	29,009,635	93.82%
heart	8,720,360,400	29,067,868	24,859,462	1,093,286	25,952,748	89.28%
liver	9,334,292,100	31,114,307	27,014,139	2,174,980	29,189,119	93.81%
Average	9,312,755,229	31,042,517	26,166,291	2,561,104	28,727,395	92.57%
Total	65,189,286,600	217,297,622	183,164,039	17,927,729	201,091,768	

**Table 2:** The sources and versions of the software used.

Software	Version	Source Link
**Augustus**	v3.3.1	https://github.com/Gaius-Augustus/Augustus accessed on 3 October 2018
**Bcftools**	v1.8.0	http://samtools.github.io/bcftools/ accessed on 28 April 2018
**blast**	v2.9	ftp://ftp.ncbi.nlm.nih.gov/blast/executables/blast+/LATEST/ accessed on 10 April 2019
**BUSCO**	3.1.0	https://busco.ezlab.org/ accessed on 3 October 2018
**BWA**	0.7.12-r1039	https://github.com/lh3/bwa accessed on 15 January 2016
**CEGMA**	v2	https://github.com/KorfLab/CEGMA_v2/ accessed on 3 October 2018
**EVidenceModeler**	v1.1.1	http://evidencemodeler.github.io/ accessed on 4 October 2018
**fastp**	0.19.4	https://github.com/OpenGene/fastp accessed on 3 October 2018
**GeMoMa**	v1.6.1	http://www.jstacs.de/index.php/GeMoMa accessed on 28 October 2019
**GMATA**	v2.2	https://sourceforge.net/projects/gmata/?source=navbar accessed on 3 October 2018
**Infernal**	v1.1.2	http://eddylab.org/infernal/ accessed on 2 October 2018
**Minimap2**	2.17(r941)	https://github.com/lh3/minimap2 accessed on 3 June 2019
**NextDenovo**	v2.0-beta.1	https://github.com/Nextomics/NextDenovo.git accessed on 20 October 2018
**NextPolish**	v1.0.5	https://github.com/Nextomics/NextPolish.git accessed on 20 October 2018
**PASA**	v2.3.3	https://github.com/PASApipeline/PASApipeline accessed on 3 October 2018
**R**	V3.5.2	https://www.r-project.org/ accessed on 2 January 2019
**RepeatMasker**	Revision 1.331	https://github.com/rmhubley/RepeatMasker accessed on 1 October 2018
**Samtools**	v1.4	https://github.com/samtools/samtools accessed on 1 May 2017
**tRNAscan-SE**	v2.0	http://lowelab.ucsc.edu/tRNAscan-SE/ accessed on 3 October 2019
**Hisat2**	v2.2.1	https://github.com/DaehwanKimLab/hisat2 accessed on 21 September 2020
**StringTie**	v2.1.4	https://github.com/gpertea/stringtie accessed on 20 July 2020
**TransDecoder**	v5.5.0	https://github.com/TransDecoder accessed on 21 September 2020
**gffread**	v0.12.3	https://github.com/gpertea/gffread accessed on 21 September 2020
**NCBI-blast**	v2.11.0	https://ftp.ncbi.nlm.nih.gov/blast accessed on 28 November 2020
**salmon**	v1.4.0	https://github.com/COMBINE-lab/salmon accessed on 28 November 2020
**MITE-Hunter**	-	https://github.com/jburnette/MITE-Hunter accessed on 3 October 2018
**RepeatModeler**	version open-1.0.11	https://github.com/Dfam-consortium/RepeatModeler accessed on 1 October 2018

**Table 3:** Summary of sequencing datasets.

Library	Number of Bases	Number of Reads	Reads Length (Mean, bp)	Reads Length (Max, bp)
nanopore-seq-lib1	45,424,703,117	2,049,727	22,161	240,976
nanopore-seq-lib2	41,854,167,498	2,031,772	20,599	243,222
Total	87,278,870,615	4,081,499	21,384	243,222
**Library**	**N50**	**>10 kb Percentage**	**>20 kb Percentage**	**>40 kb Percentage**
nanopore-seq-lib1	31,576	75.85	48.11	13.48
nanopore-seq-lib2	30,199	70.82	42.38	11.99
Total	30,943	73.35	45.26	12.74
**Library**	**Number of Bases**	**Number of Reads**	**Reads Length (Mean, bp)**	**Reads Length (Max, bp)**
NGS-Genome	54,380,742,900	362,538,286	150	150
NGS-HiC	60,291,587,700	401,943,918	150	150
NGS-RNASeq	65,189,286,600	434,595,244	150	150
	**Coverage(X) of Predicted Assembly**		**Predicted Genome Size (bp, 2*n* = 40)**	
**Nanopore-seq**	160.836		542,656,829	
**NGS-Genome**	100.212			

**Table 4:** Summary of genome assembly.

	Assembly (Preliminary)		Assembly (Polished)	
	Contig Length (bp)	Contig Number	Contig Length (bp)	Contig Number
N50	23,408,022	10	23,556,738	10
N60	20,522,422	13	20,673,841	13
N70	18,786,353	16	18,887,485	16
N80	17,637,255	18	17,756,054	18
N90	7,606,484	23	7,646,988	23
Longest	35,774,240	1	36,041,605	1
Total	539,115,043	62	542,654,729	62
Length ≥ 5 kb	539,115,043	62	542,654,729	62

## Data Availability

The genome sequence and genome annotation of *Trachidermus fasciatus* has been stored in the Genome Warehouse of National Genomics Data Center, Beijing Institute of Genomics (BIG), Chinese Academy of Sciences, under accession number GWHACFF00000000.2, which is publicly accessible at https://bigd.big.ac.cn/gwh.

## Supplementary Materials

Available on https://www.mdpi.com/article/10.3390/genes12050692/s1. Figure S1: Sashimi plot illustration of skin specific expression of novel gene Trf.20428; Figure S2: (A) Mappability of novel transcripts protein sequences. Novel transcripts derived protein sequences were mapped to NCBI NR database by Blastp. (B) Distribution of blastp identity percentage for mappable novel transcripts derived protein sequences; Figure S3: Sashimi plot of gene Trf.5711 (Transcript ID Trf.5711.1), a novel transcript with unmappable protein sequence. The exon–exon junctional linkages and exon-level reads coverages were illustrated. Table S1: Statistics about Hi-C enhanced genome assembly. Table S2: Expression of novel transcripts. Table S3. List of novel genes and transcripts. Table S4. Relationship between Hi-C assembly scaffolds and contigs of polished preliminary assembly. Supplementary File 1: Sequence of 82 predicted novel transcripts.
