# Supplementary figures and images for: Nanopore Sequencing and Hi-C Based De Novo Assembly of Trachidermus fasciatus Genome
Source: Genes (Basel). 2021 May 6;12(5):692. doi: 10.3390/genes12050692 (PMC8148166; doi:10.3390/genes12050692)

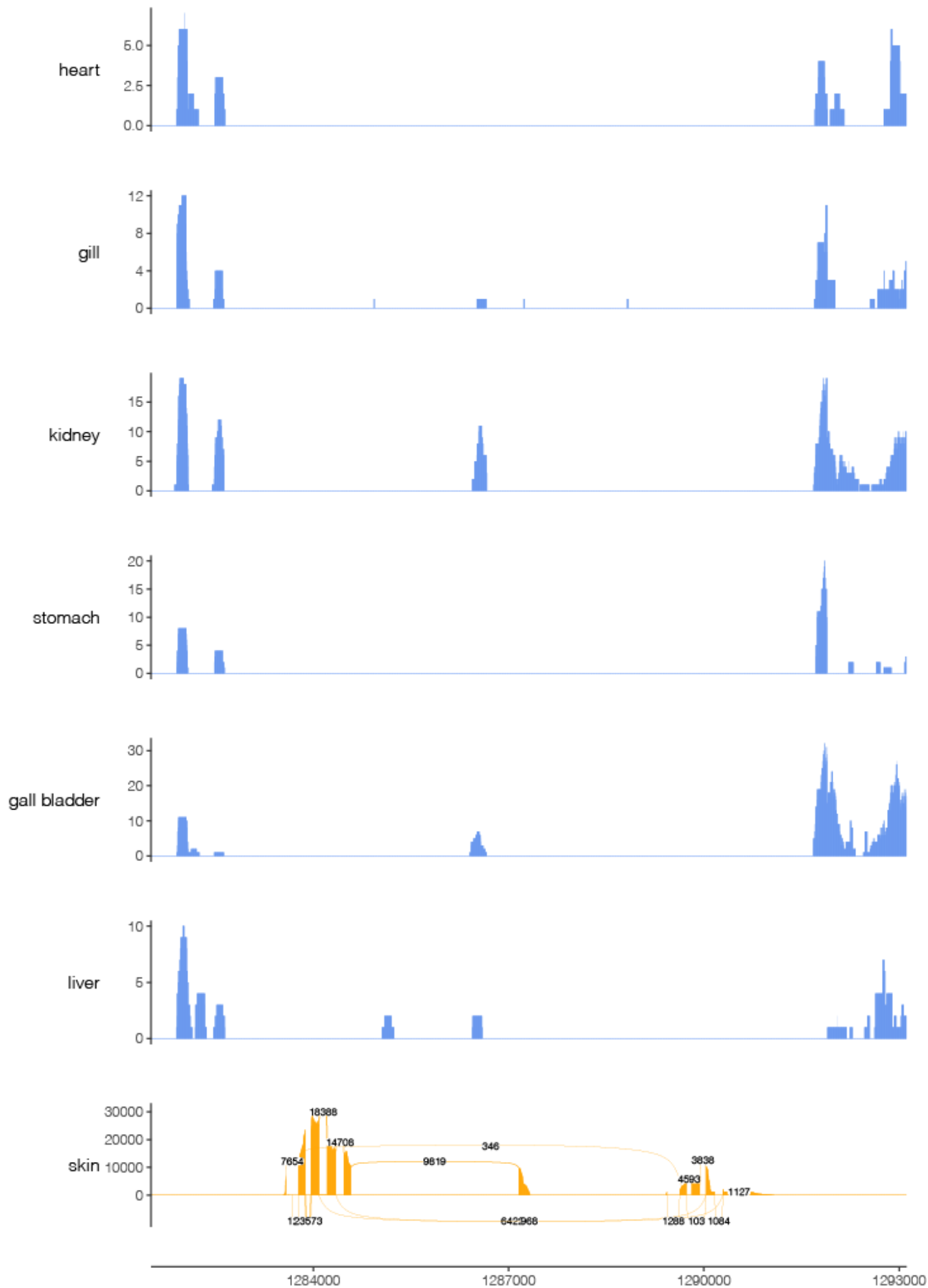

Trf.20428.3

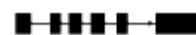

Trf.20428.2

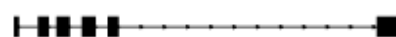

Trf.20428.1

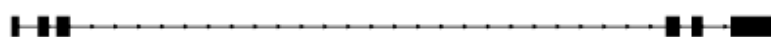

Supplement: Supplementary file 1 [file genes-12-00692-s001.zip › Figure S1 - Skin specific expressed novel gene.pdf]

A

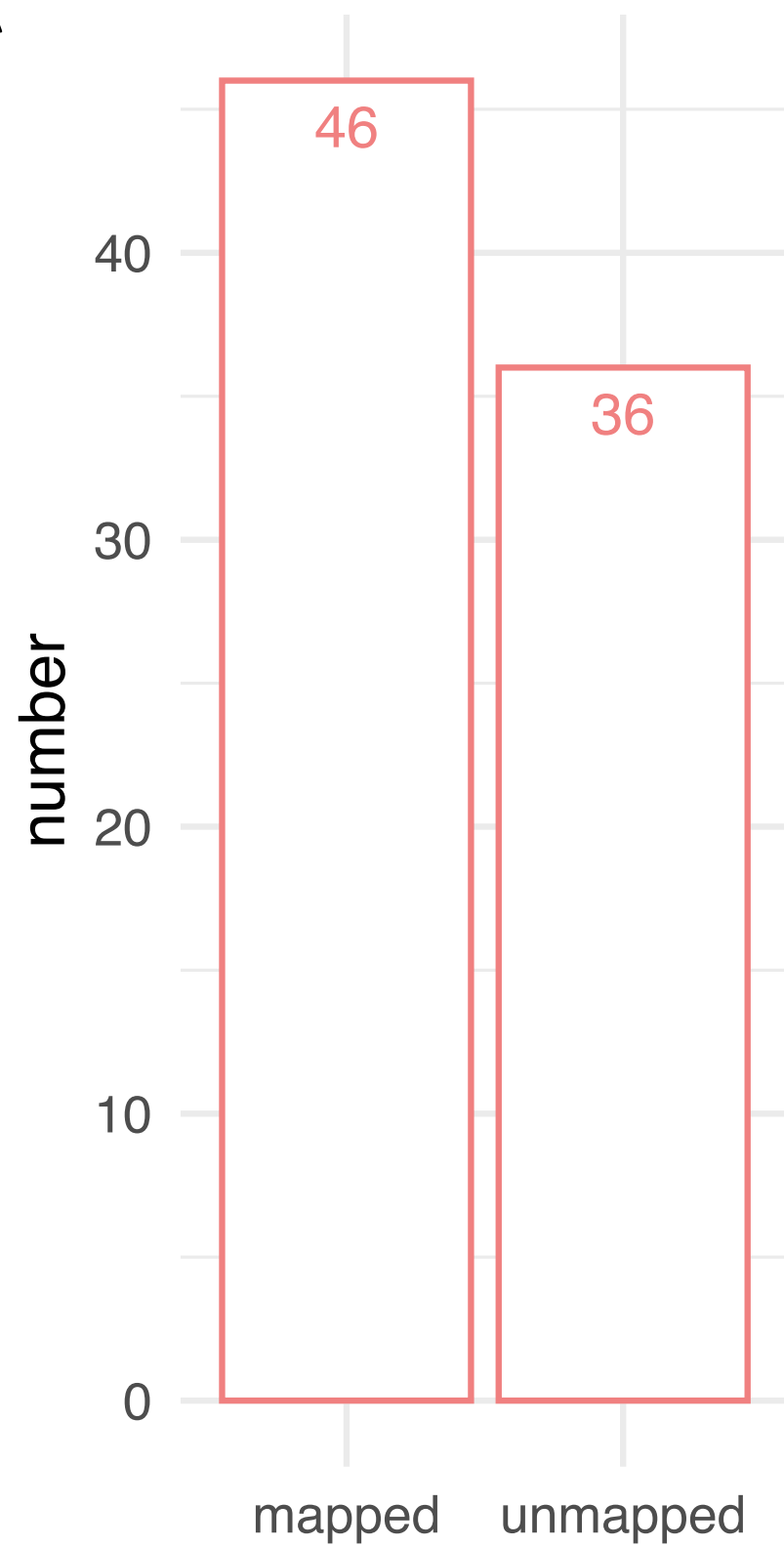

B

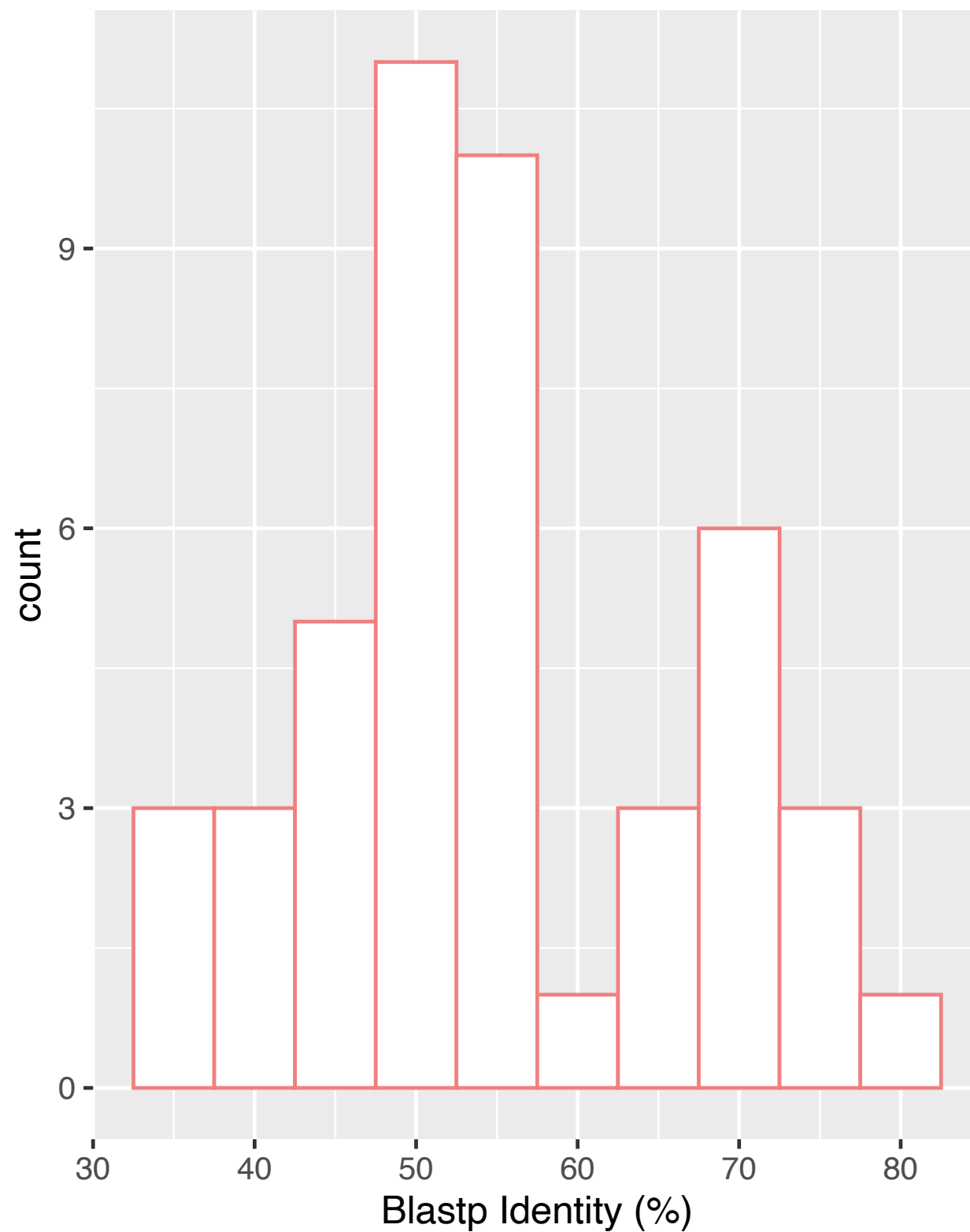

Supplement: Supplementary file 1 [file genes-12-00692-s001.zip › Figure S2-Novel gene mappability.pdf]

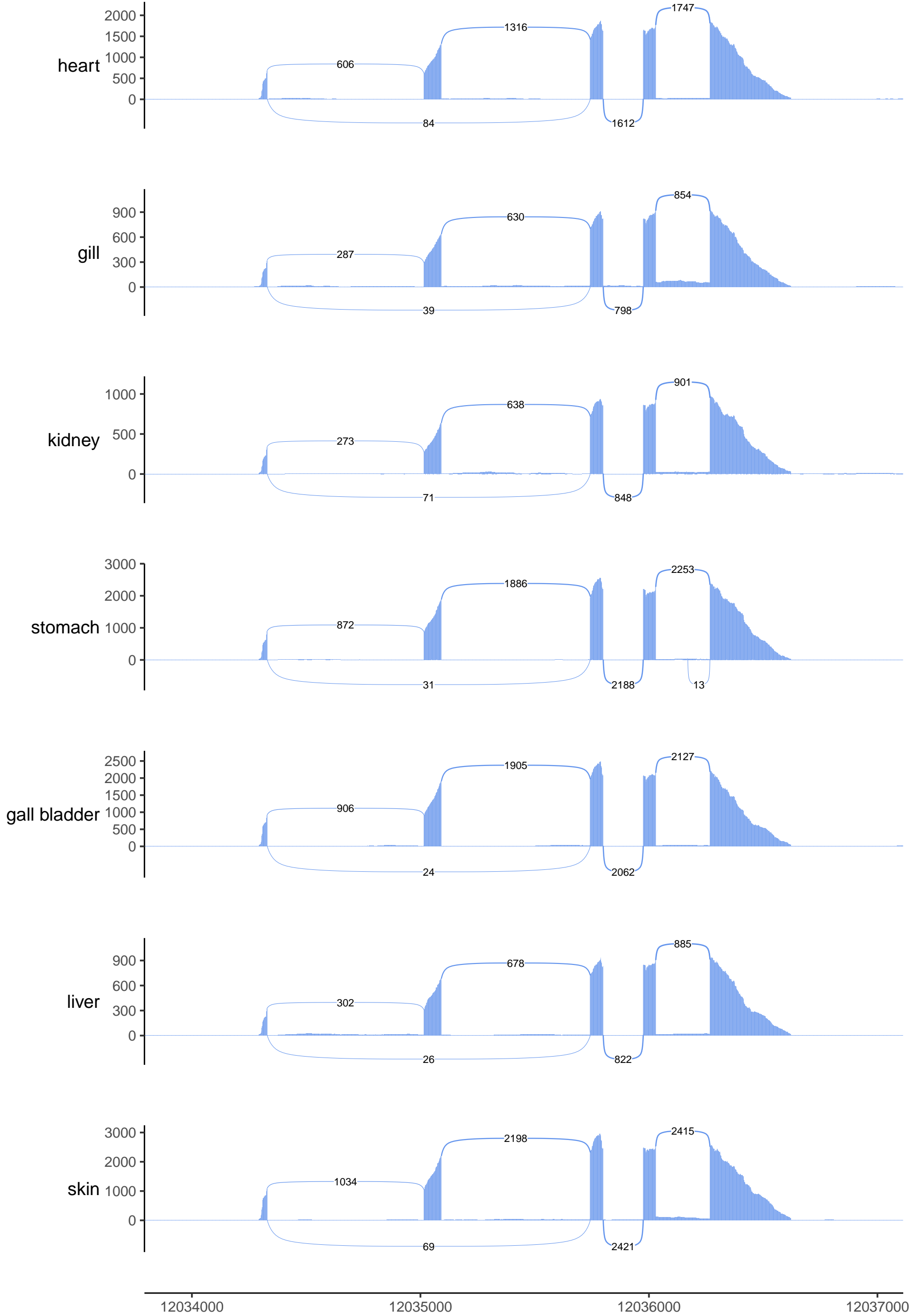

Trf.5711.1

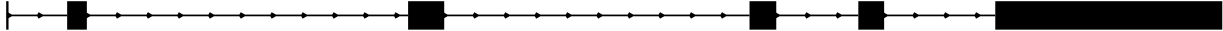

Supplement: Supplementary file 1 [file genes-12-00692-s001.zip › Figure S3 - All tissues.pdf]
